# A binuclear molybdenum oxyfluoride: μ-oxido-bis­[(2,2′-bipyrid­yl)fluoridodioxidomolybdenum(VI)]

**DOI:** 10.1107/S1600536810026383

**Published:** 2010-07-10

**Authors:** Paul DeBurgomaster, Jon Zubieta

**Affiliations:** a Department of Chemistry, Syracuse University, Syracuse, New York 13244, USA

## Abstract

The title compound, [Mo_2_F_2_O_5_(C_10_H_8_N_2_)_2_], is a centrosymmetric binuclear molybdenum(VI) species with the metal atoms in a distorted octa­hedral environment. The coordination geometries of the symmetry-equivalent molybdenum sites are defined by the *cis*-terminal oxide groups and the N-atom donors of the bipyridyl ligand in the equatorial plane with axial F and bridging O atoms. The bridging O atom occupies a center of symmetry. The mol­ecules stack in the *a*-axis direction, and the crystal packing is stabilized by weak intra- and inter­molecular C—H⋯O and C—H⋯F hydrogen bonds.

## Related literature

For oxidofluoridomolybdates and -vanadates, see: Adil *et al.* (2010 ▶); Burkholder & Zubieta (2004 ▶); Jones *et al.* (2010 ▶); Michailovski *et al.* (2006 ▶, 2009 ▶).
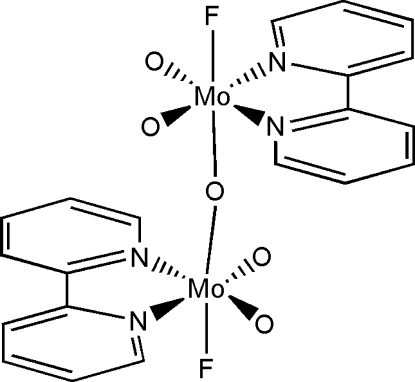


## Experimental

### 

#### Crystal data

[Mo_2_F_2_O_5_(C_10_H_8_N_2_)_2_]
*M* *_r_* = 622.25
Monoclinic, 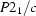

*a* = 6.9180 (4) Å
*b* = 15.6494 (8) Å
*c* = 10.4544 (5) Å
β = 108.933 (1)°
*V* = 1070.59 (10) Å^3^
*Z* = 2
Mo *K*α radiation
μ = 1.23 mm^−1^
*T* = 90 K
0.30 × 0.24 × 0.18 mm

#### Data collection

Bruker APEX CCD area-detector diffractometer
Absorption correction: multi-scan (*SADABS*; Sheldrick, 1996 ▶) *T*_min_ = 0.709, *T*_max_ = 0.809
10668 measured reflections
2647 independent reflections
2609 reflections with *I* > 2σ(*I*)
*R*_int_ = 0.025

#### Refinement

*R*[*F* ^2^ > 2σ(*F* ^2^)] = 0.049
*wR*(*F* ^2^) = 0.094
*S* = 1.39
2647 reflections
183 parameters
All H-atom parameters refined
Δρ_max_ = 0.81 e Å^−3^
Δρ_min_ = −1.47 e Å^−3^

### 

Data collection: *SMART* (Bruker, 1998 ▶); cell refinement: *SAINT* (Bruker, 1998 ▶); data reduction: *SAINT*; program(s) used to solve structure: *SHELXS97* (Sheldrick, 2008 ▶); program(s) used to refine structure: *SHELXL97* (Sheldrick, 2008 ▶); molecular graphics: *CrystalMaker* (Palmer, 2005 ▶); software used to prepare material for publication: *SHELXL97*.

## Supplementary Material

Crystal structure: contains datablocks I, global. DOI: 10.1107/S1600536810026383/om2344sup1.cif

Structure factors: contains datablocks I. DOI: 10.1107/S1600536810026383/om2344Isup2.hkl

Additional supplementary materials:  crystallographic information; 3D view; checkCIF report

## Figures and Tables

**Table 1:** Hydrogen-bond geometry (Å, °)

*D*—H⋯*A*	*D*—H	H⋯*A*	*D*⋯*A*	*D*—H⋯*A*
C1—H1⋯O1	0.95 (5)	2.56 (5)	3.104 (6)	117 (3)
C2—H2⋯F1^i^	0.92 (5)	2.39 (5)	3.201 (5)	146 (4)
C2—H2⋯F1^ii^	0.92 (5)	2.59 (5)	3.103 (5)	116 (4)
C3—H3⋯F1^ii^	0.89 (6)	2.71 (6)	3.183 (5)	115 (5)
C4—H4⋯O1^iii^	0.88 (5)	2.52 (5)	3.224 (5)	137 (4)
C7—H7⋯O1^iii^	0.90 (6)	2.42 (6)	3.263 (6)	155 (5)
C8—H8⋯F1^iv^	0.90 (6)	2.57 (6)	3.435 (5)	162 (5)
C8—H8⋯O2^iv^	0.90 (6)	2.66 (6)	3.317 (6)	130 (5)
C9—H9⋯O2^v^	0.89 (6)	2.70 (6)	3.207 (6)	117 (4)
C10—H10⋯O2^v^	0.87 (6)	2.47 (5)	3.063 (5)	126 (4)
C10—H10⋯O2	0.87 (6)	2.68 (5)	3.199 (6)	120 (4)

Symmetry codes: (i) 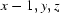; (ii) 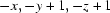; (iii) 
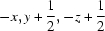
; (iv) 
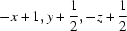
; (v) 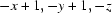.

**Fig. 1.**
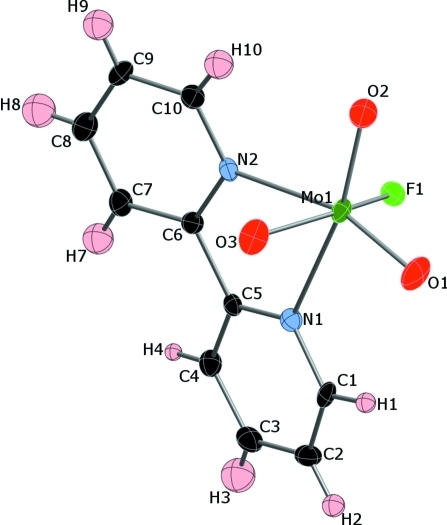
View of the asymmetric unit of the title structure, with the atom numbering scheme and the displacement ellipsoids drawn at the 50% probability level. Color scheme: molybdenum, dark green; fluorine, light green; oxygen, red; nitrogen, blue; carbon, black; hydrogen, pink.

**Fig. 2.**
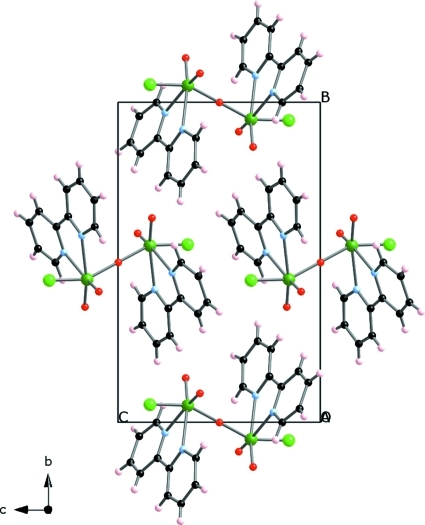
A packing diagram illustrating the stacking of binuclear units of the title compound. Color code as for Fig. 1.

## supplementary crystallographic information

### Comment

The contemporary interest in metal oxides reflects their vast compositional range and structural versatility. One area of oxide chemistry that has witnessed considerable activity is that of zeolitic materials, compositions forming open-framework structures consisting of metal oxide components and organic moieties acting as charge compensating cations, structure-directing agents or ligands. While the majority of these materials are simple oxides or oxyanion based, the introduction of fluoride to substitute for some oxo-groups provides a novel class of oxyfluorometalates (Adil *et al.*, 2010; Jones, *et al.*, 2010; Michailovski, *et al*., 2006 and 2009; Burkholder and Zubieta, 2004).

In the course of our investigations of organic-inorganic oxide hybrid materials of molybdenum and vanadium, we have noted that F^-^ is a useful mineralizing agent. However, under appropriate conditions of temperature and stoichiometry, fluoride may be incorporated into the coordinate covalent framework of the material to provide novel oxyfluorometalate composites. In the course of these investigations, the title compound [Mo_2_F_2_O_5_(bpy)_2_] was isolated.

The compound crystallizes in the monoclinic space group *P2_1_/c* with two binuclear molecules per unit cell. The bridging oxo-group sits at a center of symmetry producing equivalent molybdenum sites. The coordination geometry is distorted octahedral with *cis*-dioxo groups and the bipyridine nitrogen donors in the equatorial plane; the axial positions are occupied by a terminal fluoride and the bridging oxo-group. The Mo—O (bridging) distance of 1.8747 (4)Å is considerably longer than the Mo—O (terminal) distances of 1.705 (3)Å and 1.710 (3) Å, as anticipated. The Mo—N distances of 2.319 (3)Å and 2.341 (3)Å exhibit the elongation associated with the strong *trans*-influence of the multiply-bonded oxo-groups. As shown in Figure, the molecules stack along the *a*-axial direction. The crystal packing is stabilized by weak intra- and intermolecular C—H···O and C—H···F hydrogen bonds (Table).

### Experimental

A mixture of MoO_3_ (0.049 g, 0.34 mmol), 2,2-bipyridyl (0.316 g, 2.02 mmol), H_2_O (5.00 mL, 277.47 mmol), and HF (0.200 mL, 5.80 mmol) in the mole ratio 1.00:595:816:17.06 was stirred briefly before heating to 70 ° C for 48 hrs (initial and final pH values of 3.5 and 3.0, respectively). Pink blocks suitable for X-ray diffraction were isolated in 40 % yield. Anal. Calcd. for C_20_H_16_F_2_Mo_2_N_4_O_5_: C, 38.6; H, 2.57; N, 9.00. Found: C, 38.3; H, 2.44; N, 9.12.

### Refinement

All the hydrogen atoms were discernable in the difference electron density map and were freely refined.

### Crystal data

**Table:**

[Mo_2_F_2_O_5_(C_10_H_8_N_2_)_2_]	*F*(000) = 612
*M**_r_* = 622.25	*D*_x_ = 1.930 Mg m^−^^3^*D*_m_ = 1.91 (2) Mg m^−^^3^*D*_m_ measured by flotation
Monoclinic, *P*2_1_/*c*	Mo *K*α radiation, λ = 0.71073 Å
Hall symbol: -P 2ybc	Cell parameters from 5107 reflections
*a* = 6.9180 (4) Å	θ = 2.4–28.3°
*b* = 15.6494 (8) Å	µ = 1.23 mm^−^^1^
*c* = 10.4544 (5) Å	*T* = 90 K
β = 108.933 (1)°	Block, pink
*V* = 1070.59 (10) Å^3^	0.30 × 0.24 × 0.18 mm
*Z* = 2	

### Data collection

**Table:**

Bruker APEX CCD area-detector diffractometer	2647 independent reflections
Radiation source: fine-focus sealed tube	2609 reflections with *I* > 2σ(*I*)
graphite	*R*_int_ = 0.025
Detector resolution: 512 pixels mm^-1^	θ_max_ = 28.3°, θ_min_ = 2.4°
Phi and ω scans	*h* = −9→9
Absorption correction: multi-scan (*SADABS*; Sheldrick, 1996)	*k* = −20→20
*T*_min_ = 0.709, *T*_max_ = 0.809	*l* = −13→13
10668 measured reflections	

### Refinement

**Table:**

Refinement on *F*^2^	Primary atom site location: structure-invariant direct methods
Least-squares matrix: full	Secondary atom site location: difference Fourier map
*R*[*F*^2^ > 2σ(*F*^2^)] = 0.049	Hydrogen site location: inferred from neighbouring sites
*wR*(*F*^2^) = 0.094	All H-atom parameters refined
*S* = 1.39	*w* = 1/[σ^2^(*F*_o_^2^) + 5.6215*P*] where *P* = (*F*_o_^2^ + 2*F*_c_^2^)/3
2647 reflections	(Δ/σ)_max_ < 0.001
183 parameters	Δρ_max_ = 0.81 e Å^−^^3^
0 restraints	Δρ_min_ = −1.47 e Å^−^^3^

### Special details

**Table:**

Geometry. All e.s.d.'s (except the e.s.d. in the dihedral angle between two l.s. planes) are estimated using the full covariance matrix. The cell e.s.d.'s are taken into account individually in the estimation of e.s.d.'s in distances, angles and torsion angles; correlations between e.s.d.'s in cell parameters are only used when they are defined by crystal symmetry. An approximate (isotropic) treatment of cell e.s.d.'s is used for estimating e.s.d.'s involving l.s. planes.
Refinement. Refinement of *F*^2^ against ALL reflections. The weighted *R*-factor *wR* and goodness of fit *S* are based on *F*^2^, conventional *R*-factors *R* are based on *F*, with *F* set to zero for negative *F*^2^. The threshold expression of *F*^2^ > σ(*F*^2^) is used only for calculating *R*-factors(gt) *etc*. and is not relevant to the choice of reflections for refinement. *R*-factors based on *F*^2^ are statistically about twice as large as those based on *F*, and *R*- factors based on ALL data will be even larger.

### Fractional atomic coordinates and isotropic or equivalent isotropic displacement parameters (Å^2^)

**Table:**

	*x*	*y*	*z*	*U*_iso_*/*U*_eq_	
Mo1	0.17273 (6)	0.44676 (2)	0.15590 (4)	0.01481 (11)	
F1	0.3489 (4)	0.44354 (17)	0.3426 (3)	0.0209 (5)	
O1	0.0243 (6)	0.3616 (2)	0.1657 (4)	0.0291 (8)	
O2	0.3584 (6)	0.4088 (2)	0.0957 (4)	0.0286 (8)	
O3	0.0000	0.5000	0.0000	0.0239 (10)	
N1	−0.0007 (5)	0.5318 (2)	0.2675 (3)	0.0137 (7)	
N2	0.2982 (5)	0.5853 (2)	0.1807 (3)	0.0129 (7)	
C1	−0.1496 (6)	0.5004 (3)	0.3104 (4)	0.0151 (8)	
C2	−0.2287 (7)	0.5437 (3)	0.3968 (4)	0.0192 (9)	
C3	−0.1536 (7)	0.6243 (3)	0.4397 (5)	0.0211 (9)	
C4	−0.0038 (7)	0.6590 (3)	0.3931 (4)	0.0177 (8)	
C5	0.0695 (6)	0.6111 (3)	0.3071 (4)	0.0127 (7)	
C6	0.2238 (6)	0.6437 (3)	0.2474 (4)	0.0142 (8)	
C7	0.2838 (7)	0.7292 (3)	0.2557 (5)	0.0189 (9)	
C8	0.4185 (7)	0.7546 (3)	0.1895 (5)	0.0205 (9)	
C9	0.4902 (7)	0.6952 (3)	0.1185 (4)	0.0189 (9)	
C10	0.4269 (7)	0.6112 (3)	0.1168 (4)	0.0167 (8)	
H1	−0.197 (7)	0.445 (3)	0.277 (5)	0.011 (11)*	
H2	−0.333 (8)	0.519 (3)	0.420 (5)	0.014 (12)*	
H3	−0.194 (9)	0.653 (4)	0.499 (6)	0.032 (16)*	
H4	0.046 (7)	0.710 (3)	0.420 (5)	0.008 (11)*	
H7	0.234 (8)	0.767 (4)	0.302 (6)	0.026 (14)*	
H8	0.463 (9)	0.809 (4)	0.193 (6)	0.031 (15)*	
H9	0.573 (8)	0.710 (4)	0.073 (5)	0.025 (14)*	
H10	0.472 (8)	0.571 (4)	0.077 (5)	0.023 (14)*	

### Atomic displacement parameters (Å^2^)

**Table:**

	*U*^11^	*U*^22^	*U*^33^	*U*^12^	*U*^13^	*U*^23^
Mo1	0.02326 (19)	0.00943 (16)	0.01567 (18)	−0.00208 (14)	0.01173 (14)	−0.00183 (14)
F1	0.0205 (13)	0.0233 (13)	0.0216 (12)	0.0055 (11)	0.0105 (10)	0.0058 (11)
O1	0.042 (2)	0.0135 (15)	0.039 (2)	−0.0115 (14)	0.0229 (18)	−0.0069 (14)
O2	0.046 (2)	0.0189 (16)	0.0340 (19)	0.0046 (15)	0.0316 (18)	−0.0013 (14)
O3	0.033 (3)	0.022 (2)	0.014 (2)	−0.006 (2)	0.0024 (19)	−0.0039 (18)
N1	0.0144 (16)	0.0139 (16)	0.0122 (16)	0.0008 (13)	0.0036 (13)	0.0006 (13)
N2	0.0158 (16)	0.0108 (15)	0.0123 (16)	0.0032 (13)	0.0048 (13)	0.0014 (12)
C1	0.016 (2)	0.016 (2)	0.0116 (19)	−0.0026 (16)	0.0028 (15)	0.0050 (15)
C2	0.0159 (19)	0.025 (2)	0.019 (2)	0.0019 (17)	0.0089 (16)	0.0024 (18)
C3	0.026 (2)	0.023 (2)	0.018 (2)	0.0085 (19)	0.0124 (18)	0.0020 (18)
C4	0.022 (2)	0.0133 (19)	0.018 (2)	0.0006 (17)	0.0069 (17)	−0.0006 (16)
C5	0.0139 (18)	0.0119 (18)	0.0115 (18)	0.0024 (15)	0.0031 (15)	0.0021 (14)
C6	0.0143 (19)	0.0116 (18)	0.0168 (19)	−0.0007 (15)	0.0051 (16)	−0.0022 (15)
C7	0.023 (2)	0.0126 (19)	0.022 (2)	−0.0015 (17)	0.0085 (18)	−0.0035 (17)
C8	0.023 (2)	0.015 (2)	0.023 (2)	−0.0050 (17)	0.0061 (18)	−0.0029 (17)
C9	0.020 (2)	0.022 (2)	0.017 (2)	−0.0019 (17)	0.0091 (17)	0.0043 (17)
C10	0.020 (2)	0.0145 (19)	0.017 (2)	0.0005 (16)	0.0085 (17)	−0.0023 (16)

### Geometric parameters (Å, °)

**Table:**

Mo1—O1	1.705 (3)	C2—H2	0.92 (5)
Mo1—O2	1.710 (3)	C3—C4	1.391 (6)
Mo1—O3	1.8747 (4)	C3—H3	0.89 (6)
Mo1—F1	1.937 (3)	C4—C5	1.387 (6)
Mo1—N2	2.319 (3)	C4—H4	0.88 (5)
Mo1—N1	2.341 (3)	C5—C6	1.491 (6)
O3—Mo1^i^	1.8747 (4)	C6—C7	1.395 (6)
N1—C1	1.344 (5)	C7—C8	1.387 (6)
N1—C5	1.347 (5)	C7—H7	0.90 (6)
N2—C10	1.336 (5)	C8—C9	1.378 (6)
N2—C6	1.350 (5)	C8—H8	0.90 (6)
C1—C2	1.376 (6)	C9—C10	1.385 (6)
C1—H1	0.95 (5)	C9—H9	0.89 (6)
C2—C3	1.383 (7)	C10—H10	0.87 (6)
			
O1—Mo1—O2	106.83 (17)	C1—C2—H2	118 (3)
O1—Mo1—O3	99.97 (14)	C3—C2—H2	123 (3)
O2—Mo1—O3	100.21 (13)	C2—C3—C4	119.1 (4)
O1—Mo1—F1	96.52 (15)	C2—C3—H3	122 (4)
O2—Mo1—F1	93.42 (15)	C4—C3—H3	119 (4)
O3—Mo1—F1	154.49 (8)	C5—C4—C3	119.1 (4)
O1—Mo1—N2	159.21 (14)	C5—C4—H4	121 (3)
O2—Mo1—N2	93.84 (14)	C3—C4—H4	120 (3)
O3—Mo1—N2	77.95 (8)	N1—C5—C4	121.7 (4)
F1—Mo1—N2	79.72 (12)	N1—C5—C6	115.0 (3)
O1—Mo1—N1	89.94 (14)	C4—C5—C6	123.2 (4)
O2—Mo1—N1	161.53 (15)	N2—C6—C7	121.7 (4)
O3—Mo1—N1	84.00 (8)	N2—C6—C5	115.4 (4)
F1—Mo1—N1	76.66 (11)	C7—C6—C5	122.8 (4)
N2—Mo1—N1	69.28 (12)	C8—C7—C6	118.6 (4)
Mo1—O3—Mo1^i^	180.0	C8—C7—H7	121 (4)
C1—N1—C5	118.3 (4)	C6—C7—H7	121 (4)
C1—N1—Mo1	122.0 (3)	C9—C8—C7	119.6 (4)
C5—N1—Mo1	119.0 (3)	C9—C8—H8	119 (4)
C10—N2—C6	118.7 (4)	C7—C8—H8	122 (4)
C10—N2—Mo1	120.9 (3)	C8—C9—C10	118.6 (4)
C6—N2—Mo1	120.0 (3)	C8—C9—H9	122 (4)
N1—C1—C2	123.3 (4)	C10—C9—H9	120 (4)
N1—C1—H1	115 (3)	N2—C10—C9	122.7 (4)
C2—C1—H1	122 (3)	N2—C10—H10	115 (4)
C1—C2—C3	118.3 (4)	C9—C10—H10	122 (4)
			
O1—Mo1—N1—C1	1.4 (3)	C1—C2—C3—C4	0.8 (7)
O2—Mo1—N1—C1	−154.2 (4)	C2—C3—C4—C5	−1.4 (7)
O3—Mo1—N1—C1	101.4 (3)	C1—N1—C5—C4	1.9 (6)
F1—Mo1—N1—C1	−95.3 (3)	Mo1—N1—C5—C4	−168.8 (3)
N2—Mo1—N1—C1	−179.2 (3)	C1—N1—C5—C6	−175.8 (3)
O1—Mo1—N1—C5	171.7 (3)	Mo1—N1—C5—C6	13.5 (4)
O2—Mo1—N1—C5	16.1 (6)	C3—C4—C5—N1	0.0 (6)
O3—Mo1—N1—C5	−88.2 (3)	C3—C4—C5—C6	177.6 (4)
F1—Mo1—N1—C5	75.0 (3)	C10—N2—C6—C7	−2.5 (6)
N2—Mo1—N1—C5	−8.9 (3)	Mo1—N2—C6—C7	−175.0 (3)
O1—Mo1—N2—C10	−168.0 (4)	C10—N2—C6—C5	175.6 (4)
O2—Mo1—N2—C10	18.0 (3)	Mo1—N2—C6—C5	3.0 (5)
O3—Mo1—N2—C10	−81.7 (3)	N1—C5—C6—N2	−10.7 (5)
F1—Mo1—N2—C10	110.7 (3)	C4—C5—C6—N2	171.6 (4)
N1—Mo1—N2—C10	−169.7 (3)	N1—C5—C6—C7	167.3 (4)
O1—Mo1—N2—C6	4.3 (6)	C4—C5—C6—C7	−10.3 (7)
O2—Mo1—N2—C6	−169.7 (3)	N2—C6—C7—C8	2.1 (7)
O3—Mo1—N2—C6	90.7 (3)	C5—C6—C7—C8	−175.9 (4)
F1—Mo1—N2—C6	−76.9 (3)	C6—C7—C8—C9	−0.4 (7)
N1—Mo1—N2—C6	2.6 (3)	C7—C8—C9—C10	−0.8 (7)
C5—N1—C1—C2	−2.5 (6)	C6—N2—C10—C9	1.3 (6)
Mo1—N1—C1—C2	167.9 (3)	Mo1—N2—C10—C9	173.7 (3)
N1—C1—C2—C3	1.2 (7)	C8—C9—C10—N2	0.4 (7)

Symmetry codes: (i) −*x*, −*y*+1, −*z*.

### Hydrogen-bond geometry (Å, °)

**Table:**

*D*—H···*A*	*D*—H	H···*A*	*D*···*A*	*D*—H···*A*
C1—H1···O1	0.95 (5)	2.56 (5)	3.104 (6)	117 (3)
C2—H2···F1^ii^	0.92 (5)	2.39 (5)	3.201 (5)	146 (4)
C2—H2···F1^iii^	0.92 (5)	2.59 (5)	3.103 (5)	116 (4)
C3—H3···F1^iii^	0.89 (6)	2.71 (6)	3.183 (5)	115 (5)
C4—H4···O1^iv^	0.88 (5)	2.52 (5)	3.224 (5)	137 (4)
C7—H7···O1^iv^	0.90 (6)	2.42 (6)	3.263 (6)	155 (5)
C8—H8···F1^v^	0.90 (6)	2.57 (6)	3.435 (5)	162 (5)
C8—H8···O2^v^	0.90 (6)	2.66 (6)	3.317 (6)	130 (5)
C9—H9···O2^vi^	0.89 (6)	2.70 (6)	3.207 (6)	117 (4)
C10—H10···O2^vi^	0.87 (6)	2.47 (5)	3.063 (5)	126 (4)
C10—H10···O2	0.87 (6)	2.68 (5)	3.199 (6)	120 (4)

Symmetry codes: (ii) *x*−1, *y*, *z*; (iii) −*x*, −*y*+1, −*z*+1; (iv) −*x*, *y*+1/2, −*z*+1/2; (v) −*x*+1, *y*+1/2, −*z*+1/2; (vi) −*x*+1, −*y*+1, −*z*.
